# The Association Between Psoriasis and Cardiovascular Comorbidity: A Narrative Review: Short title: A Narrative Review on Association Between Psoriasis and Cardiovascular Comorbidity

**DOI:** 10.31661/gmj.v15i.4156

**Published:** 2026-08-27

**Authors:** Mohammad Almohideb

**Affiliations:** ^1^ Department of Dermatology, College of Medicine, King Saud bin Abdulaziz University for Health Sciences, Riyadh, Saudi Arabia; ^2^ King Abdullah International Medical Research Center, Riyadh, Saudi Arabia

**Keywords:** Psoriasis, Cardiovascular Disease, Myocardial Infarction, Stroke, Atherosclerosis, Disease Severity, Narrative Review

## Abstract

Psoriasis is a chronic immune-mediated inflammatory disease that extends beyond the skin and is increasingly recognized as a systemic disorder associated with multiple cardiometabolic comorbidities. Growing evidence indicates that patients with psoriasis, particularly those with moderate to severe disease, have an increased risk of cardiovascular morbidity and mortality, including myocardial infarction, stroke, atherosclerotic cardiovascular disease, and cardiovascular death. This narrative review summarizes the current evidence linking psoriasis with cardiovascular comorbidity, with emphasis on disease severity, systemic inflammation, psoriatic arthritis, pediatric psoriasis, and cardiovascular risk factors. The available literature suggests that mild psoriasis may not consistently increase cardiovascular event risk, whereas moderate to severe psoriasis is more frequently associated with adverse cardiovascular outcomes. Pediatric psoriasis has also been linked with obesity, hypertension, diabetes, arrhythmia, and other cardiovascular comorbidities. Shared inflammatory pathways, endothelial dysfunction, metabolic disturbances, and traditional cardiovascular risk factors appear to contribute to this association. These findings support the need to approach psoriasis as a systemic inflammatory disease requiring early cardiovascular risk assessment, lifestyle modification, and multidisciplinary management.

## Introduction

Psoriasis is one of the most common chronic inflammatory skin diseases encountered in dermatological practice. It is characterized by abnormal keratinocyte proliferation, epidermal hyperplasia, and immune-mediated inflammation involving complex interactions between innate and adaptive immune pathways [1]. Although psoriasis was historically regarded as a disease limited mainly to the skin and joints, accumulating evidence has redefined it as a systemic inflammatory disorder with important extracutaneous consequences [2]. The systemic nature of psoriasis is clinically relevant because chronic inflammation may contribute to several comorbidities, particularly cardiovascular disease. Psoriasis has been associated with reduced life expectancy, and cardiovascular complications are considered a major contributor to excess mortality in affected patients [3, 4]. In addition to physical manifestations, psoriasis has a substantial impact on mental health, quality of life, lifestyle behaviors, and treatment adherence, which may indirectly influence cardiovascular risk [5, 6]. Recent epidemiological and clinical studies have shown that patients with psoriasis have increased rates of major vascular events, including myocardial infarction, stroke, and cardiovascular death [7, 8]. Contrary to earlier assumptions that mortality in psoriasis was mainly linked to infection or renal disease, contemporary evidence suggests that cerebrovascular and cardiovascular complications represent leading causes of death in this population [9, 10]. Cardiovascular risk in psoriasis appears to be influenced by disease severity. Moderate to severe psoriasis has been more consistently associated with increased cardiovascular morbidity than mild disease [11]. Patients with psoriasis also have a higher prevalence of traditional cardiovascular risk factors, including obesity, smoking, hypertension, diabetes mellitus, dyslipidemia, and subclinical atherosclerosis [11–13]. These factors may interact with systemic inflammation to accelerate atherogenesis and increase the likelihood of adverse cardiovascular outcomes. Previous studies have reported that patients with moderate to severe psoriasis may have higher mortality and approximately five years shorter life expectancy compared with controls, largely because of cardiovascular comorbidities [14, 15]. Furthermore, psoriasis accompanied by cardiovascular disease or psoriatic arthritis may impose substantial clinical and economic burden [7, 16, 17]. This narrative review aims to synthesize current evidence regarding the association between psoriasis and cardiovascular comorbidity, with emphasis on myocardial infarction, stroke, atherosclerotic cardiovascular disease, cardiovascular mortality, disease severity, psoriatic arthritis, pediatric psoriasis, and clinical implications for screening and prevention.

**Table:** Table1. Proposed mechanisms linking psoriasis with cardiovascular comorbidity

Study	Population/context	Main cardiovascular finding
Dowlatshahi et al. [18]	Population-based Rotterdam Study	Mild psoriasis was not associated with increased atherosclerosis or incident cardiovascular events
Egeberg et al. [19]	Danish nationwide cohort	Severe psoriasis showed slightly increased myocardial infarction risk; mild psoriasis showed no clear increase
Ahlehoff et al. [20]	Danish nationwide cohort	Cardiovascular events and mortality increased with psoriasis severity
Levesque et al. [21]	Canadian retrospective cohort	Psoriasis was associated with increased myocardial infarction risk overall
Kwa et al. [22]	Pediatric hospitalized population in the United States	Pediatric psoriasis was associated with obesity, hypertension, diabetes, arrhythmia, and valvular heart disease
Jung et al. [23]	Korean nationwide population-based cohort	Psoriasis was independently associated with increased atherosclerotic cardiovascular disease risk

**Table:** Table2. Objectives and outcomes of the included studies

Author / Year	Aim	Design	Lesion Type / Guidance	Diagnostic Performance	Radio-Pathologic Concordance / Outcomes
Jordan et al., 2022 [6]	Review CT-guided CNB for head and neck masses	Retrospective cohort	Deep cervical masses / CT-guided	Sensitivity 94%, Specificity 97%, Accuracy 95%	Concordance 89%; no major complications
Pan et al., 2025 [11]	Assess CT-guided CNB for deep suprahyoid lesions	Prospective cohort	Deep suprahyoid spaces / CT-guided	Sensitivity 90%, Specificity 95%, Accuracy 92%	Concordance 87–90%; factors affecting diagnostic failure analyzed
Serra-García et al., 2022 [12]	Evaluate diagnostic accuracy of image-guided biopsies	Multicenter retrospective	Head and neck lesions / US and CT-guided	Sensitivity 90–95%, Specificity 92–97%	Concordance 88–92%; some inconclusive tests reported
Tipaldi et al., 2022 [13]	Develop scoring system for biopsy outcome prediction	Retrospective study	Lesions with variable imaging features / CT-guided	Sensitivity 91%, Specificity 94%, Accuracy 92%	Concordance 87–90%; imaging features influence outcomes
Avritscher et al., 2010 [14]	CT-guided PNB of hilar lymph nodes	Retrospective study	Pulmonary hilar lymph nodes / CT-guided PNB	Sensitivity 91.4%, Accuracy 92.8%	Pneumothorax 48%, thoracostomy tube 32%; PNB is viable alternative to EUS/bronchial FNAB
Hillen et al., 2020 [15]	CT-guided CNB of head and neck masses	Retrospective study	Head and neck masses / CT-guided CNB	Diagnostic sample rate 100%	Concordant histopathologic diagnosis 93%; 1 minor complication (small hematoma)
Vogl et al., 2024 [16]	CT-guided CNB of head and neck tumors	Retrospective study	Head and neck masses / CT-guided CNB	Diagnostic yield 90.4%	False negative rate 2.7%, 9 puncture-related complications (5.7%), no reinterventions needed
Wang et al., 2022 [17]	paramaxillary CT-guided FNA of head and neck lesions	Retrospective study	Head and neck lesions / CT-guided FNA (paramaxillary)	Diagnostic yield 85%	100% concordance in diagnostic FNAs; no postprocedural complications
Wu et al., 2013 [18], untreated patients	Evaluate efficacy of CT-guided CNB for deep head and neck tumors in untreated patients	Retrospective study	Deep suprahyoid / CT-guided CNB	Diagnostic yield 90% (9/10)	Adequate specimens in all; no complications; 1 false negative for meningioma
Wu et al., 2013 [19]	Evaluate efficacy of CT-guided CNB for deep suprahyoid lesions in treated patients	Retrospective study	Deep suprahyoid / CT-guided CNB	Diagnostic accuracy 96.4%	1 false negative (atypia); 2 minor complications (hematoma, transient facial palsy); no difference between 18G and 20G needles

### Psoriasis as a Systemic Inflammatory Disease

Psoriasis is driven by a complex inflammatory network involving keratinocytes, dendritic cells, T lymphocytes, neutrophils, and multiple cytokine pathways [9]. The immune dysregulation underlying psoriasis is not restricted to the skin; rather, it may influence systemic immune and vascular function [1, 2]. This systemic inflammatory burden provides a biological basis for the association between psoriasis and cardiometabolic disease [9]. Several inflammatory mediators implicated in psoriasis, including tumor necrosis factor-alpha, interleukin-17, interleukin-23, and other proinflammatory cytokines, may also contribute to endothelial dysfunction, insulin resistance, vascular inflammation, and atherosclerotic plaque development. As a result, psoriasis may share mechanistic pathways with atherosclerosis and metabolic syndrome [7, 11]. The recognition of psoriasis as a systemic disease has changed clinical practice [9]. Dermatological assessment alone may be insufficient, particularly in patients with extensive skin involvement, long disease duration, systemic inflammation, or psoriatic arthritis. Instead, psoriasis should be viewed as a chronic inflammatory disorder requiring broader evaluation for metabolic and cardiovascular risk [10, 11].

### Cardiovascular Risk Factors in Psoriasis

Patients with psoriasis have higher rates of several cardiovascular risk factors compared with the general population. These include smoking, obesity, hypertension, diabetes mellitus, dyslipidemia, and subclinical atherosclerosis [11–12]. Some of these risk factors may be behavioral or lifestyle-related, while others may be directly influenced by systemic inflammation [10, 11, 12]. Obesity is particularly important because adipose tissue produces proinflammatory cytokines that may worsen psoriasis and increase cardiovascular risk [10]. Similarly, insulin resistance and diabetes may contribute to vascular damage and atherogenesis. Hypertension and dyslipidemia further compound cardiovascular risk, especially in patients with moderate to severe psoriasis [10, 11]. The relationship between psoriasis and cardiovascular risk factors is likely bidirectional [13]. Systemic inflammation may promote metabolic dysfunction, while obesity and metabolic syndrome may intensify inflammatory pathways involved in psoriasis. This overlap supports the need for integrated management targeting both cutaneous disease activity and cardiovascular risk reduction [10, 11, 12, 13].

### Psoriasis Severity and Cardiovascular Outcomes

Disease severity is one of the most important modifiers of cardiovascular risk in psoriasis. Evidence suggests that mild psoriasis is not always associated with a significant increase in cardiovascular events, whereas moderate to severe psoriasis is more consistently linked with adverse cardiovascular outcomes [18–23]. Dowlatshahi et al. [18] evaluated patients in the Rotterdam Study and found that individuals with predominantly mild psoriasis did not have increased atherosclerosis or incident cardiovascular events compared with those without psoriasis [18]. In this study, carotid intima-media thickness, ankle-brachial index, pulse-wave velocity, and coronary artery calcium scores were not significantly different between psoriasis patients and controls. The adjusted hazard ratio for incident cardiovascular disease was not increased, suggesting that mild psoriasis alone may not substantially elevate cardiovascular risk in a general population setting [18]. In contrast, Ahlehoff et al. [20] reported that psoriasis was associated with increased all-cause mortality and adverse cardiovascular events, with risk rising according to disease severity [20]. The rate ratio for a composite endpoint of myocardial infarction and stroke was higher in severe psoriasis than in mild psoriasis. Cardiovascular mortality also increased with severity, supporting the concept of a dose-response relationship between inflammatory skin burden and cardiovascular outcomes [20]. Similarly, Egeberg et al. [19] found that mild psoriasis was not associated with a clearly increased risk of myocardial infarction, whereas severe psoriasis was associated with a modestly elevated risk [19]. These findings suggest that cardiovascular risk may be concentrated among patients with more severe inflammatory disease, although the magnitude of risk may vary according to age, comorbidities, treatment exposure, and study design [19]. Levesque et al. [21] reported an increased risk of myocardial infarction among patients with psoriasis in a Canadian cohort [21]. Although the adjusted hazard ratio was significantly increased for psoriasis overall, the association differed between mild and severe disease categories. This variation highlights the complexity of using treatment-based definitions of severity and the potential influence of confounding variables. Jung et al. [23] provided further evidence from a Korean nationwide cohort, showing that psoriasis was independently associated with increased risk of atherosclerotic cardiovascular disease [23]. In this study, patients with moderate to severe psoriasis had a two- to three-fold increased risk of myocardial infarction, and women with moderate to severe psoriasis had increased risk of ischemic stroke [23]. Taken together, these findings suggest that cardiovascular risk in psoriasis is not uniform. Severity, systemic inflammation, age, sex, psoriatic arthritis, and traditional cardiovascular risk factors all influence the degree of risk [23] (Table1).

### Myocardial Infarction in Psoriasis

Myocardial infarction is among the most studied cardiovascular outcomes in psoriasis. Several cohort studies have reported that psoriasis, particularly moderate to severe disease, is associated with increased myocardial infarction risk [19–21, 23]. Egeberg et al. found that the adjusted hazard ratio for myocardial infarction was not significantly increased in mild psoriasis but was modestly increased in severe psoriasis [19]. Their findings also suggested that the relationship between psoriasis and myocardial infarction may vary by age and by the presence or absence of psoriatic arthritis. Ahlehoff et al. [20] observed that cardiovascular event rates increased with psoriasis severity and decreased with increasing age of onset [20]. This suggests that younger patients with severe psoriasis may represent a particularly important high-risk group. The finding is clinically significant because younger patients may otherwise be classified as low risk using standard cardiovascular risk calculators [20]. Levesque et al. [21] also reported a higher relative risk of myocardial infarction in patients with psoriasis [21]. However, differences between mild and severe categories were not always consistent, possibly because severity was defined using treatment exposure. Patients receiving systemic therapy may differ from untreated patients in ways that are not fully captured by administrative datasets [21]. Jung et al. [23] reported elevated myocardial infarction risk in both men and women with moderate to severe psoriasis, with particularly high hazard ratios in women [23]. This sex-specific variation suggests that cardiovascular risk assessment in psoriasis should account for both disease severity and demographic factors [23].

### Stroke and Atherosclerotic Cardiovascular Disease

Stroke and atherosclerotic cardiovascular disease are also important outcomes in psoriasis. Chronic systemic inflammation may accelerate atherosclerosis, promote endothelial dysfunction, and contribute to plaque instability, thereby increasing the risk of ischemic vascular events [7, 24]. Ahlehoff et al. reported increased risks of stroke and composite cardiovascular endpoints among patients with psoriasis, particularly those with severe disease [20]. Jung et al.[23] further demonstrated that psoriasis was associated with increased risk of atherosclerotic cardiovascular disease, including ischemic heart disease [23]. In their cohort, women with moderate to severe psoriasis had increased risk of ischemic stroke [23]. These findings support the hypothesis that psoriasis may contribute to vascular disease through both direct inflammatory mechanisms and indirect effects mediated by traditional cardiovascular risk factors. However, the degree of association varies across studies, and mild psoriasis may not independently increase stroke risk in all populations [18].

### Psoriatic Arthritis and Cardiovascular Comorbidity

Psoriatic arthritis is an inflammatory musculoskeletal manifestation of psoriatic disease and may further increase systemic inflammatory burden. Patients with psoriatic arthritis often have higher rates of obesity, metabolic syndrome, hypertension, diabetes, and cardiovascular disease compared with the general population [16, 17]. Ahlehoff et al. [20] reported that patients with psoriatic arthritis and those with severe skin involvement alone had comparable cardiovascular risks. This suggests that both joint inflammation and extensive cutaneous inflammation may contribute to adverse cardiovascular outcomes [20]. The coexistence of psoriasis and psoriatic arthritis may also complicate management, as these patients often require systemic therapy and may have higher healthcare utilization [20]. Cardiovascular screening is therefore particularly important in this subgroup, especially when other risk factors such as obesity, hypertension, or diabetes are present [20, 23].

### Pediatric Psoriasis and Cardiovascular Risk

Although most cardiovascular outcome studies focus on adults, pediatric psoriasis is also associated with cardiometabolic comorbidities. Kwa et al. [22] examined hospitalized children in the United States and found that pediatric psoriasis was associated with increased odds of several cardiovascular comorbidities [22]. These included obesity, hypertension, diabetes, arrhythmia, valvular heart disease, and lipid abnormalities [22]. Obesity was the most frequent cardiovascular comorbidity among hospitalized children with psoriasis, occurring more commonly than in controls. After adjustment for age, sex, and race, psoriasis remained significantly associated with obesity, hypertension, diabetes, arrhythmia, and valvular heart disease [22]. These findings are important because pediatric psoriasis may identify a group of children at risk for early cardiometabolic abnormalities. Early detection of obesity, hypertension, dyslipidemia, and glucose abnormalities may help reduce long-term cardiovascular risk [10, 22]. However, pediatric evidence remains limited, and more prospective studies are needed to determine whether childhood psoriasis independently predicts adult cardiovascular events [22].

### Mechanisms Linking Psoriasis and Cardiovascular Disease

Several mechanisms may explain the relationship between psoriasis and cardiovascular disease. The most widely accepted explanation is shared systemic inflammation. Psoriasis and atherosclerosis both involve immune activation, cytokine release, endothelial dysfunction, and inflammatory cell recruitment [7, 24]. Inflammatory cytokines may promote endothelial activation, oxidative stress, insulin resistance, and lipid abnormalities. These processes contribute to atherogenesis and may increase the risk of plaque rupture, thrombosis, myocardial infarction, and stroke. Severe psoriasis may represent a state of greater inflammatory burden, explaining why cardiovascular risk appears more pronounced in moderate to severe disease [20, 23]. Traditional cardiovascular risk factors also play a major role. Patients with psoriasis have higher rates of smoking, obesity, hypertension, diabetes, and dyslipidemia [11, 12, 13]. These factors may partly mediate the association between psoriasis and cardiovascular outcomes. However, some studies suggest that psoriasis remains independently associated with cardiovascular disease even after adjustment for conventional risk factors [20, 23]. Treatment may also influence cardiovascular risk. Systemic anti-inflammatory therapies may theoretically reduce vascular inflammation, while some treatments may affect blood pressure, lipids, or metabolic parameters. However, evidence remains insufficient to determine the precise cardiovascular impact of individual psoriasis therapies in routine clinical practice [24, 25] (Table2).

**Table:** Table2. Proposed mechanisms linking psoriasis with cardiovascular comorbidity

Mechanism	Potential cardiovascular effect
Chronic systemic inflammation	Promotes endothelial dysfunction and vascular inflammation
Cytokine activation	Contributes to insulin resistance, oxidative stress, and atherogenesis
Obesity and metabolic syndrome	Amplify inflammation and increase cardiometabolic risk
Hypertension and dyslipidemia	Accelerate atherosclerosis and vascular injury
Psoriatic arthritis	Adds systemic inflammatory burden
Severe skin disease	Reflects greater inflammatory load and higher cardiovascular risk
Lifestyle factors	Smoking, inactivity, and psychological stress may worsen cardiovascular outcomes

### Clinical Implications

The available evidence supports the need to approach psoriasis as more than a dermatological disorder, but rather as a chronic systemic inflammatory disease with important cardiometabolic implications [3, 5]. Patients with psoriasis, particularly those with moderate-to-severe disease or psoriatic arthritis, should undergo cardiovascular risk assessment as part of routine clinical care [16, 17, 24]. Screening should include blood pressure measurement, body mass index assessment, lipid profiling, glucose testing, smoking history, and evaluation of family history and lifestyle-related cardiovascular risk factors [5, 10, 11]. Early lifestyle modification should be emphasized as a core component of management. Weight reduction, smoking cessation, regular physical activity, a healthy diet, and optimal control of hypertension, diabetes, and dyslipidemia may reduce cardiovascular risk and may also contribute to improvement in psoriasis severity [10, 11, 13]. Multidisciplinary care is essential for comprehensive management. Dermatologists, primary care physicians, cardiologists, rheumatologists, and endocrinologists may all contribute to risk assessment, prevention, and long-term care [8, 17]. This approach is particularly important for patients with severe psoriasis, psoriatic arthritis, pediatric psoriasis associated with obesity or hypertension, and patients requiring systemic therapy [22, 24]. Standard cardiovascular risk calculators may underestimate risk in younger patients with severe psoriasis because they do not fully capture the contribution of chronic systemic inflammation [3, 5, 8]. Therefore, clinicians should maintain a low threshold for cardiovascular screening, preventive counseling, and risk-factor modification in high-risk patients with psoriasis [24].

### Strengths and Limitations of the Evidence

The current evidence base has several strengths. Many included studies were large population-based or registry-based investigations with substantial sample sizes and broad geographic representation, including Europe, North America, and Asia [18, 19, 20, 21, 22]. Several studies used national databases, diagnostic coding systems, and adjustment for major cardiovascular risk factors, which improves the reliability and generalizability of their findings [20, 21, 22, 23]. However, important limitations remain. Definitions of psoriasis severity vary widely across studies. Some investigations classify severe psoriasis according to systemic therapy use or hospital-based treatment, which may not accurately reflect actual skin disease severity [18, 19, 20, 21]. Residual confounding related to smoking, obesity, dyslipidemia, diabetes, physical inactivity, and treatment exposure may also influence the observed associations [21, 23]. In addition, follow-up duration differs across studies, and some pediatric data are cross-sectional rather than longitudinal [22]. Cardiovascular endpoints also vary among studies and include myocardial infarction, ischemic heart disease, stroke, heart failure, cardiovascular mortality, atrial fibrillation, and composite cardiovascular outcomes [25, 26]. This heterogeneity limits direct comparison across studies. Moreover, although many studies demonstrate consistent associations between psoriasis and cardiovascular disease, causality remains difficult to establish because observational designs cannot fully eliminate residual confounding [23, 25].

### Future Directions

Future research should prioritize large prospective multicenter studies using standardized definitions of psoriasis severity and cardiovascular outcomes. These studies should carefully adjust for lifestyle factors, metabolic comorbidities, systemic inflammatory burden, treatment exposure, and the presence of psoriatic arthritis [24, 25]. There is also a need to develop cardiovascular risk prediction tools specifically tailored to patients with psoriasis [24]. Such tools should account for disease severity, disease duration, systemic inflammation, psoriatic arthritis, metabolic syndrome, and treatment history [16, 17]. Pediatric studies are particularly needed to determine whether childhood psoriasis predicts long-term cardiovascular morbidity and mortality [22]. Further genetic, molecular, and immunological studies may clarify the shared inflammatory pathways linking psoriasis and atherosclerosis [7, 8]. A better understanding of these mechanisms may support the development of therapeutic strategies that reduce both cutaneous inflammation and cardiovascular risk [24].

## Conclusion

Psoriasis is a chronic systemic inflammatory disease associated with increased cardiovascular morbidity, particularly in patients with moderate to severe disease. Evidence links psoriasis with myocardial infarction, stroke, atherosclerotic cardiovascular disease, cardiovascular mortality, and multiple cardiometabolic risk factors.

The association appears to be influenced by disease severity, age, sex, psoriatic arthritis, systemic inflammation, and traditional cardiovascular risk factors. Mild psoriasis may not consistently increase cardiovascular event risk, whereas moderate to severe psoriasis is more frequently associated with adverse cardiovascular outcomes.Pediatric psoriasis is also associated with obesity, hypertension, diabetes, arrhythmia, and other cardiovascular comorbidities.

These findings emphasize the importance of early cardiovascular risk screening, preventive lifestyle counseling, and multidisciplinary care in patients with psoriasis. Recognizing psoriasis as a systemic inflammatory disease may improve long-term outcomes and reduce cardiovascular morbidity and mortality.

## Conflict of Interest

The author declares that there is no conflict of interest related to this manuscript.

## AI Disclosure Statement

During the preparation of this manuscript, AI–based language assistance (ChatGPT) was used to support manuscript restructuring, language polishing, grammar correction, and improvement of clarity and flow. The authors reviewed, edited, and approved all AI-assisted content and take full responsibility for the accuracy, integrity, and final version of the manuscript.
